# Supplementary material for: Identification and functional analysis of non-coding regulatory small RNA FenSr3 in Bacillus amyloliquefaciens LPB-18
Source: PeerJ. 2023 May 15;11:e15236. doi: 10.7717/peerj.15236 (PMC10194069; doi:10.7717/peerj.15236)
Supplement: Supplemental Information 4 [file peerj-11-15236-s004.zip › KO/CK-vs-T1_map/map04392.html]

KEGG PATHWAY: Hippo signaling pathway - multiple species - Reference pathway


|  |  |
| --- | --- |
| **Hippo signaling pathway - multiple species - Reference pathway** |  |

[
Pathway menu
| Organism menu
| Pathway entry
| Show description
| User data mapping
]

Hippo signaling pathways control diverse aspects of cell proliferation, survival, and morphogenesis in eukaryotes. The core organization of these networks is conserved over a billion years of evolution, with related forms described in animals and fungi. In Drosophila and mammals, Hippo/MST co-operate with Mats/Mob1 and Salvador/WW45 to activate Warts/LATS, which negatively regulates Yorkie/YAP. Yorkie/YAP interact with Scalloped/TEAD to promote gene transcriptions and control organ size through the balance between cell proliferation and apoptosis. In C. elegans, WTS-1 YAP-1 EGL-44 axis is conserved and regulates thermotolerance and healthy lifespan. In S. cerevisiae, the LATS-related Dbf2 or Dbf20 kinase in complex with Mob1 controls mitotic exit and cytokinesis, and the Hippo/MST family of kinases, STE-20, modulates Tec1, the putative yeast TEAD ortholog.

|  |  |  |
| --- | --- | --- |
| Reference pathway | 184% 150% 122% 100% 82% 67% 55% | 图片下载 |
